# Mesenchymal Stem Cells Enhance Nerve Regeneration in a Rat Sciatic Nerve Repair and Hindlimb Transplant Model

**DOI:** 10.1038/srep31306

**Published:** 2016-08-11

**Authors:** Damon S. Cooney, Eric G. Wimmers, Zuhaib Ibrahim, Johanna Grahammer, Joani M. Christensen, Gabriel A. Brat, Lehao W. Wu, Karim A. Sarhane, Joseph Lopez, Christoph Wallner, Georg J. Furtmüller, Nance Yuan, John Pang, Kakali Sarkar, W. P. Andrew Lee, Gerald Brandacher

**Affiliations:** 1 Department of Plastic and Reconstructive Surgery, Vascularized Composite Allotransplantation (VCA) Laboratory, Johns Hopkins University School of Medicine, Baltimore, Maryland, USA

## Abstract

This study investigates the efficacy of local and intravenous mesenchymal stem cell (MSC) administration to augment neuroregeneration in both a sciatic nerve cut-and-repair and rat hindlimb transplant model. Bone marrow-derived MSCs were harvested and purified from Brown-Norway (BN) rats. Sciatic nerve transections and repairs were performed in three groups of Lewis (LEW) rats: negative controls (n = 4), local MSCs (epineural) injection (n = 4), and systemic MSCs (intravenous) injection (n = 4). Syngeneic (LEW-LEW) (n = 4) and allogeneic (BN-LEW) (n = 4) hindlimb transplants were performed and assessed for neuroregeneration after local or systemic MSC treatment. Rats undergoing sciatic nerve cut-and-repair and treated with either local or systemic injection of MSCs had significant improvement in the speed of recovery of compound muscle action potential amplitudes and axon counts when compared with negative controls. Similarly, rats undergoing allogeneic hindlimb transplants treated with local injection of MSCs exhibited significantly increased axon counts. Similarly, systemic MSC treatment resulted in improved nerve regeneration following allogeneic hindlimb transplants. Systemic administration had a more pronounced effect on electromotor recovery while local injection was more effective at increasing fiber counts, suggesting different targets of action. Local and systemic MSC injections significantly improve the pace and degree of nerve regeneration after nerve injury and hindlimb transplantation.

For more than 50 years, surgeons have performed increasingly complex repairs of injured nerves^1^,^2^. Despite improvements in surgical techniques, outcomes have been disappointing. As a result, increasing emphasis has been placed on promoting and delineating nerve regeneration. The increasing use of reconstructive transplantation to treat devastating injuries of the face and extremities has further demonstrated the need for improvements in this area. While functional outcomes from reconstructive transplantation are limited by the immunological consequences of allotransplantation such as rejection, nerve regeneration presents additional challenges. Cellular therapies, particularly mesenchymal stem cells (MSCs), present an attractive treatment option for enhancing nerve regeneration.

Mesenchymal stem cells (MSCs) have great translational potential in regenerative medicine given their availability and potential for multilineage differentiation into bone, cartilage, muscle, fat, and tendon^3^,^4^. Many studies have demonstrated the ability of MSCs to repair tissue defects and injuries throughout the body and to promote healing by production of growth factors, cytokines, and adhesion molecules^5^,^6^. Additionally, MSCs have been shown to improve nerve regeneration both peripherally and centrally. Early studies have demonstrated the ability of MSCs to differentiate into Schwann cell-like cells^7^. Since Schwann cells promote functional and histological central and peripheral nerve regeneration^8^,^9^,^10^, the possibility of MSCs to replace difficult to obtain Schwann cells has been an area of active investigation. Additionally, studies have found that MSCs can improve nerve regeneration through the production of local neurotrophic factors for induction of axonal growth^11^ and direct conversion of stem cells into myelinating cell lines^2^,^12^. From this basic premise, MSCs have been used alone and as a component of various engineered bioconstructs to enhance nerve regeneration. Finally, numerous studies have shown that injecting MSCs into an injured nerve can improve both functional and histological regeneration^2^,^13^,^14^,^15^. In a peripheral nerve crush model, systemic injection of MSCs promoted nerve regeneration by a theorized mode of paracrine induction of axonal growth^16^,^17^.

However, the regenerative properties of MSCs only form part of their appeal. These cells also have immunologic features that make them an attractive addition to the reconstructive transplant setting. MSCs lack expression of HLA-DR (major histocompatibility complex class II antigen), which renders them significantly less immunogenic than other cell types^18^,^19^. MSCs have also been shown to reduce inflammation *in vivo* by inhibiting production of pro-inflammatory cytokines and stimulating production of anti-inflammatory cytokines and antigen-specific T-regulatory cells^20^,^21^. Their ability to suppress alloreactive T cell proliferation allows MSCs to be transplanted across MHC barriers without stimulating an immune response^22^. Furthermore, preclinical and clinical trials utilizing allogeneic MSCs in transplantation have shown that these cells effectively mitigate acute graft-versus-host disease in mice and humans^23^,^24^,^25^,^26^,^27^,^28^. Lastly, MSCs have the unique capability of migrating to areas of hypoxia or tissue injury, and therefore augmenting tissue repair, limiting apoptosis, and promoting angiogenesis^19^,^29^,^30^ demonstrated by studies using renal, cardiac, and bowel allografts^31^,^32^,^33^.

These characteristics make MSCs an attractive choice for systemic (intravenous) administration. Systemic application allows MSCs to migrate to areas of pathology that otherwise would be difficult to treat medically or surgically, such as areas affected by neurodegenerative disease, osteoarthritis, or steroid-resistant graft-versus-host disease^23^,^30^,^34^. These homing properties have been demonstrated in rat models of myocardial infarction in which intravenous administration of MSCs localized to infarcted tissue, whereas non-infarcted rats showed MSC-homing to the bone marrow^35^. Similarly, Gruenloh *et al.*^34^ used a hindlimb ischemia model to demonstrate that MSCs could home to areas of hypoxic injury^34^.

The translational potential of MSCs is high, particularly since they have already been FDA-approved for use in human trials^36^,^37^,^38^,^39^. They are relatively safe when compared to stem cells of embryonic origin, which have more tumor-forming potential. Of the several animal and human studies using systemic administration of allogeneic MSCs, none have shown any tumor formation or other adverse effects after transplantation^34^,^40^,^41^. Furthermore, numerous studies have examined their application in peripheral nerve regeneration enhancement^13^,^42^,^43^,^44^. Dezawa *et al.* in 2001 were the first group to successfully induce MSCs into functional schwann cells from bone-marrow derived mesenchymal stem cells^2^. Using cytokine stimulation, Dezawa and others^45^,^46^ demonstrated that MSC treatment could augment nerve regeneration in both small and large animal models. In addition to bone marrow derived MSCs, adipose- or umbilical-derived stem cells have also been investigated, and have been found to be effective sources of MSCs for the treatment of peripheral nerve injuries^47^,^48^,^49^. More recently, olfactory and hair follicle stem cells have been found to be efficient sources for cellular therapy to improve peripheral nerve regeneration in various animal models^50^,^51^,^52^,^53^. These studies have demonstrated that hair follicle stem cells can transdifferentiate into Schwann cells and enhance peripheral nerve regeneration and restore nerve function. With less malignant potential and produced more efficiently, hair follicle pluripotent stem cells have high regenerative potential for spinal cord and peripheral nerve regeneration^50^. Similarly, olfactory have been found to improve peripheral nerve regeneration but unlike other sources, displays unique neurogenic characteristic that might make it ideally suited for cellular therapy^52^. All these studies have demonstrated that MSCs can enhance peripheral nerve regeneration via two mechanisms: 1) MSCs differentiate into functional schwann cells, therefore providing myelination and axon sprouting support; 2) MSCs change the inflammatory environment at the nerve co-aptation site facilitating a more regenerative rather than intraneural scar formation state^16^. These properties make MSCs treatment as a highly attractive option to augment peripheral nerve regeneration.

The ability of MSCs to promote tissue regeneration, decrease inflammation, and differentiate into Schwann cells in specialized *in vitro* conditions^7^ support the hypothesis that these cells may positively influence nerve regeneration outcomes in the setting of limb transplantation. The homing capabilities of MSCs, together with their immunomodulatory properties and regenerative potential, make them an attractive choice for targeted clinical therapies to alter the cytokine milieu or exert immunomodulatory effects, specifically at sites of tissue damage. Thus, MSCs have great potential for improving outcomes in regenerative medicine and vascularized composite allotransplantation (VCA).

Despite their great potential, many unanswered questions regarding the use of MSCs remain. Although MSCs have shown great potential in enhancing peripheral nerve regeneration in small and large animal models, its potential has not been tested clinically. In fact, MSC treatment for peripheral nerve injuries was only recently explored in a preclinical, primate model^13^. Furthermore, the potential for MSC cellular therapy in VCA has not been fully explored. Therefore, the aims of this study were: (1) to investigate whether or not the administration of bone marrow-derived mesenchymal stem cells (BM-MSCs) could improve nerve regeneration in a sciatic nerve cut-and-repair model; (2) to assess the efficacy of BM-MSCs to improve nerve generation in a reconstructive transplantation model, and (3) to examine whether systemic or direct application of BM-MSCs provides superior functional outcomes in the above models. We hope that our findings informs translational opportunities to improve nerve recovery outcomes following reconstructive transplantation.

## Results

### Characterization and Immunomodulatory Effect of BM-MSCs

Flow cytometric analysis of MSCs revealed consistent and homogenous expression of CD29 and CD90 and no expression of CD11, CD45, RT1A, and RT1B (Supplementary Figure 1). Moreover, culture of BM-MSCs in cell-specific differentiation media demonstrated that isolated MSCs possessed chondrogenic, osteogenic, and adipogenic properties (Supplementary Figure 2).

### Sciatic Nerve Transection and Repair

**Figure 1:**
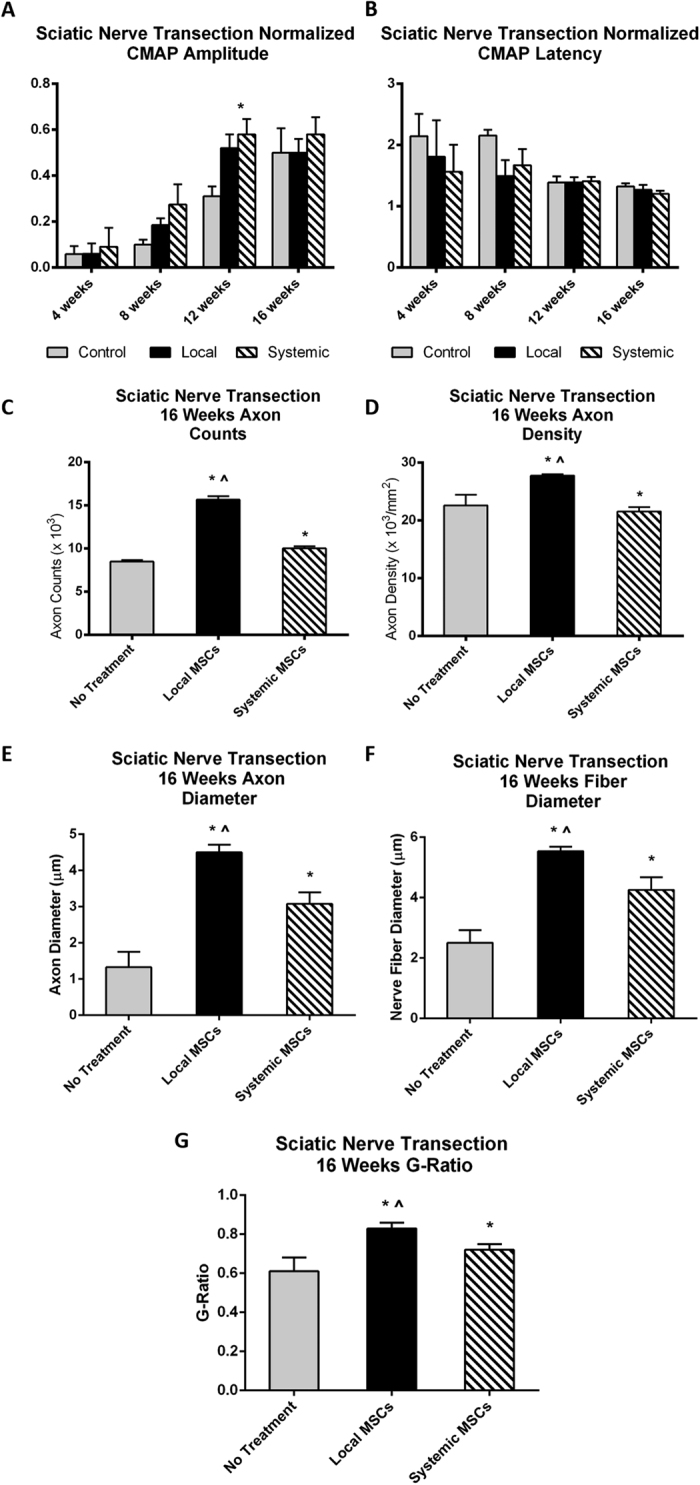
Sciatic nerve transection and repair. (**A**) Normalized experimental CMAP amplitudes are significantly higher in local and systemic MSC treatment groups compared to control at the 12 week time point (*p < 0.05). (**B**) Latency fell over time in a manner consistent and similar across all groups. (**C**) Mean axon count and (**D**) axon density demonstrated significant improvement in local and systemic MSC treatment groups compared to no treatment controls. (**E**) Axon diameter, (**F**) nerve fiber diameter, and (**G**) G-ratio demonstrated significant improvement in local and systemic MSC treatment groups when compared to the no treatment controls. (*significantly different from control, ^significant difference between local and systemic MSC; p < 0.05). Error bars represent standard deviation. CMAP=compound muscle action potential, Local=local MSC injection, Systemic=systemic MSC injection.

Compound muscle action potentials were recorded at the level of the foot following sciatic nerve transection and repair. Immediately following transection and repair all signal was lost (as expected); all animals recovered detectible action potentials over the next 6 to 16 weeks. Both local and systemic MSC treatment resulted in significantly higher CMAP normalized amplitude ratios at weeks 8 and 12 compared to controls (0.58 ± 0.13 and 0. 52 ± 0.11 vs. 0.31 ± 0.11 at week 12; mean ± SD; p < 0.05) (Fig. 1A). By 16 weeks, control animals recovered to levels similar to both experimental groups. In contrast, no specific patterns were seen in latency measurements although latency did decrease over time in a consistent and similar manner across all groups (Fig. 1B). The mean fiber number and fiber density at a point 5–8 mm distal to the nerve coaptation site was assessed to determine nerve regeneration. Standardized nerve histomorphometry techniques were used. These studies demonstrated that by week 16 both the local and systemic MSC treatment groups had higher mean axon counts per nerve than controls (local MSCs = 15,676 and systemic MSCs = 10,052 vs. controls = 8,501; p < 0.05) (Fig. 1C). The local MSC group also demonstrated a trend towards significantly higher axon density than controls (Fig. 1D). Similar findings were seen when assessing axon and nerve fiber diameter (Fig. 1E,F). More importantly, the G-ratio was statistically significantly higher in the local and systemic MSC treatment groups when compared to controls (local MSC = 0.83 and systemic MSC = 0.72 vs controls 0.61; p < 0.05) (Fig. 1G). Supplementary Figure 3 illustrates representative images from the experimental groups stained with Toluidine blue. Computer assisted gait-based analysis using the CatWalk system was performed and although results followed a similar trend, they did not demonstrate any statistically significant differences due to high variability in both control and experimental groups (data not shown) in animal posture or paw placement and position.

### Syngeneic Hindlimb Transplantation

**Figure 2:**
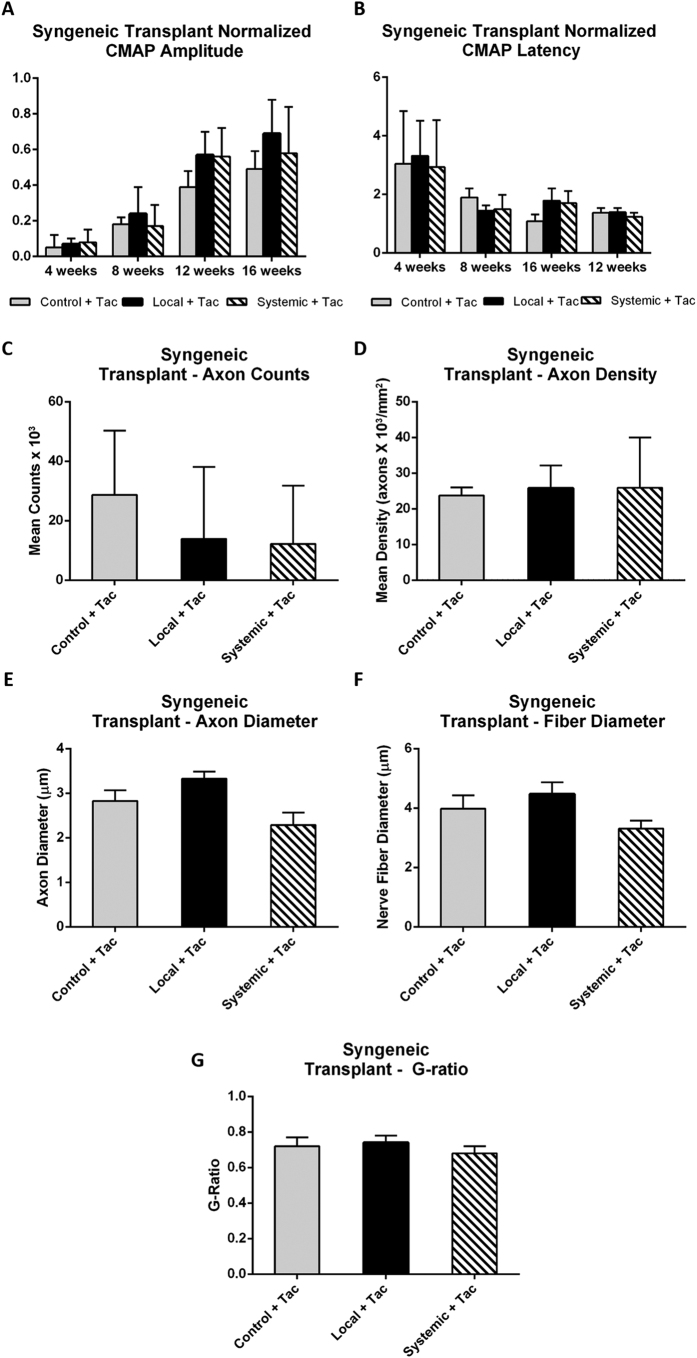
Syngeneic hindlimb transplant. (**A**) Normalized experimental CMAP amplitudes were similar between local and systemic MSC treatment groups when compared to control at the 12 week time point. (**B**) Latency fell over time in a consistent manner and was similar across all groups. (**C**) Mean axon count and (**D**) axon density were similar across local and systemic MSC treatment groups when compared to no treatment controls. (**E**) Axon diameter, (**F**) nerve fiber diameter, and (**G**) G-ratio were also similar between in local and systemic MSC treatment groups when compared to the no treatment controls. (*significantly different from control, ^significant difference between local and systemic MSC; p < 0.05). Error bars represent standard deviation. CMAP=compound muscle action potential, Local=local MSC injection, Systemic=systemic MSC injection.

Building on the results from the sciatic nerve transection groups, we sought to determine if a similar effect could be detected in animals undergoing hindlimb transplantation. A syngeneic transplant model (Lew to Lew) was first used to isolate the effect of MSCs independent of an alloimmune response. Animals underwent electromyography (EMG), histomorphometry and gait analysis outcomes testing similar to that performed in the nerve transection/repair groups. In electrophysiological testing, rats receiving syngeneic hindlimb transplants had recovery curves with shapes similar to those receiving sciatic nerve repairs. There was improvement in all groups over all time points. Mean normalized CMAP amplitudes at 16 weeks in the tacrolimus-only control, local MSC treatment, and systemic MSC treatment groups were 0.49 ± 0.1, 0.69 ± 0.19, and 0.58 ± 0.26, respectively (Fig. 2A,B). Nerve histomorphometry data failed to demonstrate any significant differences in axonal counts and density for local and systemic MSC treatment groups when compared to controls (Fig. 2C,D). Additionally, there were no statistical significant differences in axon diameter, fiber diameter or G-ratio for local and systemic MSC treatment groups when compared to controls (Fig. 2E–G). Computer assisted gait-based analysis using the CatWalk system was also performed and failed to demonstrate any differences in dynamic as well as static gait parameters between groups such as animal posture or paw placement/position. The lack of detectable functional differences using the CatWalk system confirms previous findings from our laboratory suggesting that unlike in other procedures such as the sciatic nerve crush injury model in which the CatWalk system has been successfully been used, the transplant procedure itself can be a significant confounder due to imperfect bone alignment and muscle group adaptation that results in a procedural bias and unreliable and inconsistent CatWalk results^54^,^55^.

### Allogeneic Hindlimb Transplantation

**Figure 3:**
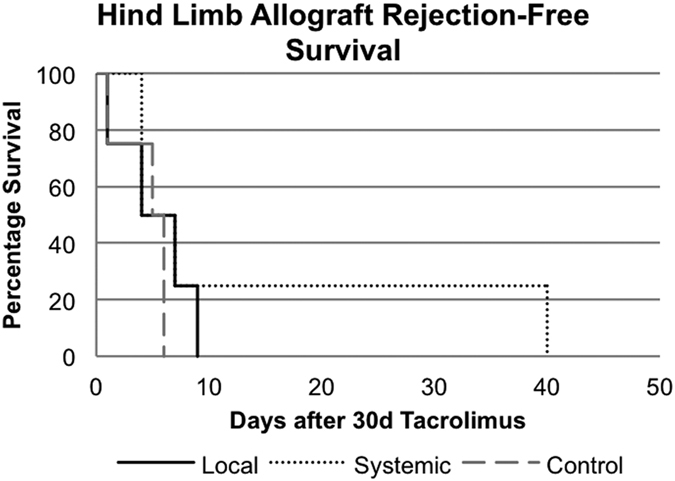
Allogeneic hindlimb transplant Kaplan-Meier Survival Curve. Rejection-free survival of skin component of allograft following local and systemic MSC treatment compared to treatment with tacrolimus alone.

No significant effect on allograft survival was found in allogeneic transplants in either the local or systemic MSC treatment groups (Fig. 3). Mean survival was 4.5, 5.25, and 13.75 days in the control, local MSC injection, and systemic MSC injection groups, respectively. The prolongation of the systemic administration group was not statistically significant as it was due to a single animal surviving for 40 days after removal of tacrolimus treatment without clinical signs of rejection. However, all other transplanted limbs in the group began to show signs of allograft rejection immediately following cessation of the 30-day course of tacrolimus similar to controls.

**Figure 4:**
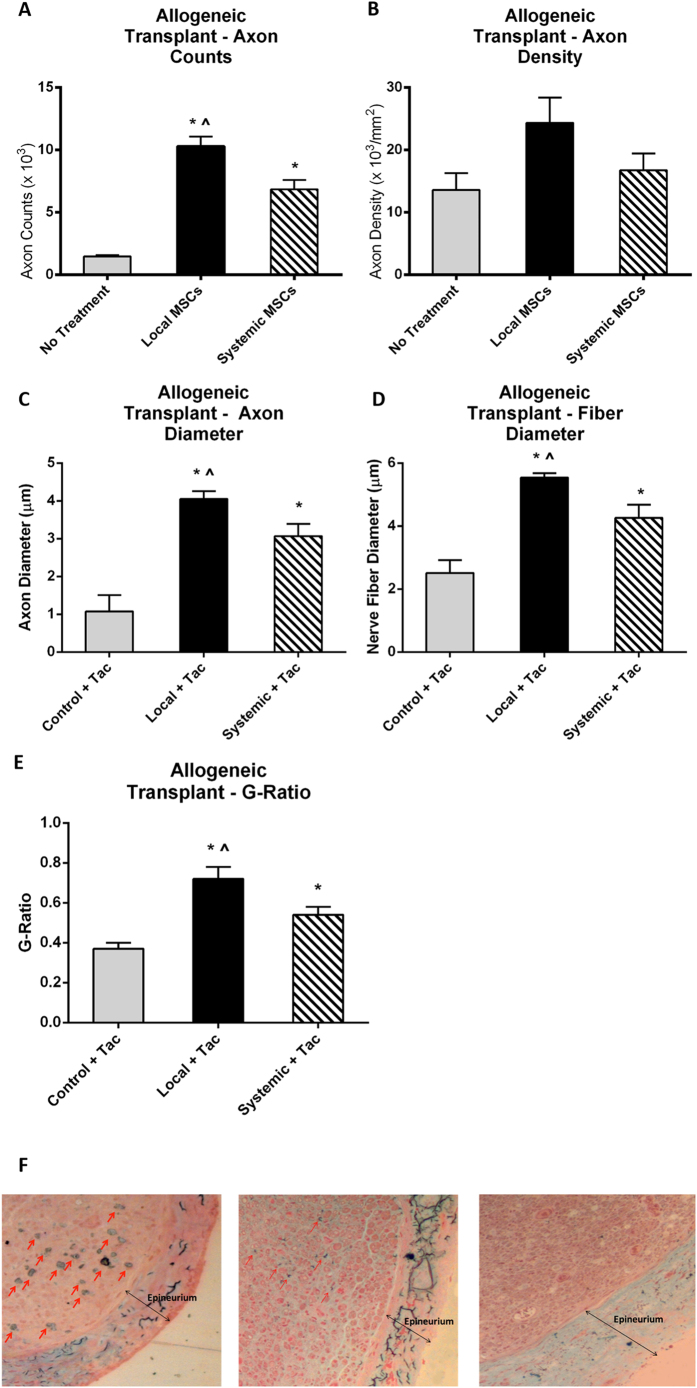
Allogeneic hindlimb transplant. (**A**) Mean axon count and (**B**) axon density demonstrated significant improvement in both the local and systemic MSC treatment groups compared to no treatment controls. (**C**) Axon diameter, (**D**) nerve fiber diameter, and (**E**) G-ratio demonstrated significant improvement in local and systemic MSC treatment groups when compared to the no treatment controls. (**F**) Masson’s trichrome staining demonstrated greater intra-neural collagen deposition (single-headed arrows) in the control tacrolimus-only group (left) as compared to systemic (middle) and local MSC treatment (right) groups. (*significantly different from control, ^significant difference between local and systemic MSC; p < 0.05). Double-headed arrows indicate the epineurium. Error bars represent standard deviation. Local=local MSC injection, Systemic=systemic MSC injection, Tac=tacrolimus.

Since graft rejection occurred prior to distal motor target reinnervation, functional outcomes such as EMG or gait analysis could not be assessed. Histomorphometric analysis at 4 weeks demonstrated that both the local MSC and systemic MSC groups exhibited significantly higher axon counts compared to controls (local MSCs = 10,289 and systemic MSCs = 6,861 vs. controls = 1,465; p < 0.05) (Fig. 4A). While the local MSC administration group also demonstrated an increase in axon density, this difference did not reach statistical significance and no difference was seen in the axon density between control and systemic administration animals (Fig. 4B). Similar findings were seen when assessing axon and nerve fiber diameter (Fig. 4C,D). More importantly, the G-ratio was statistically significantly higher in the local and systemic MSC treatment groups when compared to controls (local MSC = 0.72 and systemic MSC = 0.54 vs controls 0.37; p < 0.05) (Fig. 4E).

To examine the possible mechanism underlying the differences in axon counts, Masson’s trichrome stain was performed to assess the amount of collagen deposition as evidence of fibrosis. Nerves were sectioned in cross-section at 4 weeks following allogeneic hindlimb transplantation. Staining of the nerve 5 mm distal to the anastomotic site confirmed that rats treated with local MSCs exhibited less endoneural collagen deposition compared to control rats (Fig. 4F), suggesting local MSC treatment dampens the inflammatory reaction solicited by the alloimmune response.

## Discussion

With more than 200 clinical VCA recipients world-wide^56^, devising ways to improve nerve regeneration is becoming increasingly important to the further expansion of this treatment modality. Over the past decade, VCA has emerged as a viable approach to tissue replacement in patients with complex tissue defects (i.e., blast/burn wounds, traumatic limb loss) not currently amenable to conventional reconstruction. Outcomes for VCA have been encouraging, demonstrating improved functional outcomes, reduced patient morbidity, and near-normal tissue restoration over time^56^,^57^,^58^. However, there is still much room for improvement. Augmenting functional outcomes is still necessary to not only improve the risk-benefit ratio of this revolutionary treatment modality, but also to improve a recipient’s quality of life. Without improving peripheral nerve regeneration, these composite tissue allografts are at risk of being not functional, therefore, rendering this non-life saving treatment option too high a risk. This study explores the potential of MSCs to promote accelerated nerve regeneration and enhanced allograft survival in an orthotopic hindlimb transplant model. The efficacy of MSC treatment was compared between two parallel experimental groups that either received systemic or local injections of MSCs.

The current study demonstrates that local injection and to a lesser degree systemic administration of bone marrow-derived MSCs can improve nerve regeneration in three animal models. The goal of these three models was to assess the nerve regenerative effects of these cells in different settings of nerve injury and repair. Our findings show that MSCs improved nerve regeneration in all three scenarios, but may not demonstrate any immunomodulatory effects *in vivo* (at least not with the chosen treatment regimen) since MSCs did not affect the clinical course of allogeneic rejection.

As expected, the control groups from all three nerve injury models demonstrated improvements in CMAPs over all time points. By comparison, the experimental groups from all three nerve injury models showed an improvement in nerve regeneration evidenced by CMAP amplitude and histomorphometry, although not all studies achieved statistical significance. Both the sciatic nerve and syngeneic transplant models showed a measurable return of electrophysiological function at 8 weeks in contrast to controls. From weeks 8–16, electrophysiological function continued to increase in both controls and experimental animals. Histomorphometric data also reflected this trend. Local and systemic MSC groups tended to have higher total nerve counts and axonal density relative to controls. In these experiments, while both local and systemic MSC injection improved nerve regeneration relative to controls, histomorphometric data showed improved axonal density among the local injection group while systemic injection was superior in the speed of electrophysiological recovery. These differential effects may be due to the location of administration. Concentrating the effect of the MSCs in the distal stump may increase the number of regenerating axons by either increasing the neurotrophic factors secreted by the system or decreasing inflammation and removing negative regulators’ of neurite outgrowth. In contrast, systemic administration may result in MSCs migrating to more distal sites along the nerve such as the neuromuscular junction. Although this is just a hypothesis, we hope to combine both local and systemic MSC administration in future studies and assess the mechanisms behind the observed differences mentioned above.

Our studies demonstrated that local or systemic MSC treatment accelerated the functional recovery and increased the number of nerve fibers distal to the site of repair. EMG recovery plateaued in all groups at a lower level than the contralateral uninjured nerve demonstrating incomplete recovery in all groups. Although the acceleration of reinnervation and electromotor recovery did not result in major differences in some of the outcome metrics, it is important to note that the innervation distances in this model are quite short. In the human clinical scenario, even modest increases in the speed of reinnervation may have an important impact on the ultimate recovery. Given the longer reinnervation distances and recovery intervals, humans may still stand to benefit from this treatment modality.

To better understand the mechanism behind the results from our local injection groups, we performed Masson’s trichrome staining of the excised distal nerve stump to identify collagen deposition, a sign of fibrosis and inflammation during nerve regeneration. Local injection of MSCs led to reduced fibrosis of the nerve when compared to the systemic MSC injection group and controls. This is consistent with recent studies that have shown the ability of MSCs to treat inflammatory conditions^59^,^60^,^61^,^62^. Several lines of evidence indicate that MSCs can alter the outcome of an ongoing inflammatory response by shifting the cytokine profile of T-cell subsets to an anti-inflammatory phenotype (decreasing TNF-α, INF-γ, IL-12 and increasing IL-4 and IL-10). This has been elegantly demonstrated in animal models of lung injury in which administration of MSCs (by both intravenous and intratracheal infusions) curbed the severe inflammatory response by mitigating pro-inflammatory networks and enhancing anti-inflammatory signals, significantly attenuating lung injury^63^,^64^,^65^. Other studies corroborated these results have revealed the central role for MSCs in mitigating pro-inflammatory networks and amplifying anti-inflammatory signals^63^,^66^,^67^.

In addition to MSCs enhancing peripheral nerve regeneration in this VCA model, immunosuppression may have also amplified nerve regeneration. Various studies have shown that tacrolimus, also known as FK506, can augment peripheral nerve regeneration^68^,^69^,^70^. Although tacrolimus alone failed to enhance peripheral nerve regeneration when compared to local or systemic administration of MSC with tacrolimus in our allogeneic model, one cannot ignore the potential incremental benefit that this immunosuppression agent may have had on the efficacy of MSC treatment. Moreover, our model did not control for the intrinsic superior regenerative capability of rats which may also impacted our findings. In fact, one may argue that the lack of a detectable difference in electrophysiological results at the study endpoint was due to murine regenerative capacity.

In this study, we have confirmed the anti-inflammatory properties of mesenchymal stem cells as demonstrated in previous studies. Although MSCs may have immunomodulatory potential, no significant effect on hindlimb allotransplantation was noted in our study (Fig. 4). Despite this, we did see a decrease in collagen deposition in these animals suggesting some an anti-inflammatory effect. By reducing the inflammatory milieu in the distal nerve fiber during regeneration, the MSCs may have reduced fibrosis and therefore, increased the nerve fiber count and accelerated functional recovery. In summary, our data provide support for further exploration of MSC cellular therapy to improve nerve regeneration and immunomodulation in vascularized composite allotransplantation.

## Methods

### Isolation and Characterization of Bone Marrow-Derived MSCs

Bone marrow-derived MSCs (BM-MSCs) were harvested from the femurs and tibias of euthanized adult Lewis (LEW) and Brown Norway (BN) rats (4–6 weeks old). BM-MSCs were isolated based on their inherent plastic adherence when grown in culture media consisting of DMEM/F12 with 10% heat-inactivated fetal bovine serum, 1% penicillin/streptomycin and 1% Fungizone (unless otherwise mentioned, all products obtained from Gibco, Life Technologies, Carlsbad, California). After 90% confluence, cells were stained with Sytox-Blue (Life Sciences, Carlsbad, California), and for CD29-FITC, CD90-PE, CD45-PerCP, and CD11b/c-AlexaFluor645 (BioLegend, San Diego, California). Labeled cells were sorted based on being CD29 and CD90 double positive and Sytox-Blue, CD45, and CD11b/c triple negative. Immunophenotypic characterization was performed by flow cytometric analysis for the aforementioned cell surface markers plus RT1A and RT1B (rat major histocompatibility complex antigens). The culture-grown BM-MSCs were tested for their ability to differentiate into adipocytes, chondrocytes, and osteoblasts using the StemPro Adipogenesis Differentiation Kit (A10070-01), Chondrogenesis Differentiation Kit (A10071-01), and hyClone AdvanceSTEM Osteogenic Differentiation Medium (SH30881, Thermo Scientific, Waltham, Massachusetts), respectively. Adipocytes were identified by oil-red O staining, chondrocytes by Alcian blue staining, and osteoblasts by von Kossa staining. For all experiments described the cells were used between passages 3–5. No differences were seen in characterization parameters between these passage numbers.

### Animals

A total of fifty-two adult Lewis rats (300 g, Charles Rivers Laboratories) and eight Brown-Norway rats (350 g, Charles River Laboratories) were used for this study. This study was designed and carried out in accordance with the Guide for the Care and Use of Laboratory Animals of the National Institutes of Health. This protocol was approved by the Johns Hopkins University Animal Care and Use Committee (RA11M130). All animals were housed in a central animal care facility with a 12-hour light/12-hour dark cycle, and provided with adequate food/water ad libitum.

### Surgical Models

#### Sciatic Nerve Transection and Repair

Twelve animals underwent sciatic nerve transection and suture repair. These animals were divided into three treatment groups: 1) a negative control group receiving no MSCs (n = 4), 2) a local injection group treated with MSCs via injection into the epineurium of the distal nerve stump (n = 4), and 3) a systemic (intravenous, or IV) group treated with an IV injection of MSCs (n = 4). Sixteen weeks was used as the end-point in all groups (Table 1).

All sciatic nerve transection and repair procedures were standardized and performed by one single highly experienced microsurgeon under aseptic conditions with the aid of an operating microscope (Leica M525 F40 surgical microscope). Of note, the microsurgeon was blinded with regard to the groupings of the rats. All animals were anesthetized with isoflurane using a VetEquip anesthesia machine (VetEquip Inc). The animal was placed in a right lateral position. A gluteal skin incision was made from the sciatic notch to a point proximal to the knee joint. The gluteal muscles were separated to expose the sciatic nerve from the sciatic notch to the point of bifurcation. The nerve was sharply transected at 1 cm proximal to the knee and re-approximated with four interrupted epineurial 10-0 nylon suture using microscopic visualization. All animals in the local injection groups received 5 × 10^4^ MSCs (reconstituted to a 5 μl volume) injected into the epineurium of the distal nerve stump using a 33-gauge blunt tip needle and custom made 50 μl plunger syringe. All animals in the systemic groups received 1 × 10^6^ MSCs injected into the penile vein at the end of the nerve transection procedure. After wound closure, all rats received buprenorphine 0.05 mg/kg SQ and were monitored post-operatively for signs of infection or distress.

#### Orthotopic Hindlimb Transplantation

Animals undergoing syngeneic hindlimb transplants and animals undergoing allogeneic hindlimb transplants were each divided into four groups: 1) a no treatment control group (n = 4); 2) a short-term immunosuppression control group (0.5 mg/kg daily tacrolimus for 30 days) (n = 4); 3) a “local” group (5 × 10^4^ MSCs injected into distal nerve stump with tacrolimus for 30 days) (n = 4); and 4) a “systemic” group (single dose of 1 × 10^6^ MSCs administered IV intra-operatively with tacrolimus for 30 days) (n = 4) (see Table 2). Tacrolimus was administered in one control group and both MSC groups to provide a consistent comparison between the syngeneic and allogeneic transplant groups.

Orthotopic hindlimb transplants were standardized and performed from LEW to LEW rats (syngeneic transplant) or BN to LEW rats (allogeneic transplant) by a single highly, experienced microsurgeon under aseptic conditions with the aid of an operating microscope (Leica M525 F40 surgical microscope). Of note, the microsurgeon was blinded with regard to the groupings of the rats. In brief, the femoral nerve, artery, and vein were isolated and divided ensuring adequate length for subsequent anastomoses. The remaining thigh muscle groups as well as the sciatic nerve were transected to expose the mid-portion of the femur. A transverse osteotomy was performed through the femur to complete allograft harvest. The recipient animal was prepared in a similar fashion. Transplantation of the allograft was performed beginning with osteosynthesis of the femur. The femoral vein and then femoral artery were anastomosed. The sciatic as well as the femoral nerve were approximated with four interrupted epineurial 10-0 nylon sutures. The sciatic nerve was transected 1 cm proximal to the knee joint in all animals. The ventral and dorsal muscle groups were then repaired with 4-0 polyglactin suture prior to skin closure with 4-0 nylon suture. The cell delivery method is the same as that described for the sciatic nerve injury model. The post-operative care was similar to that described for the sciatic nerve injury model as well. Animals receiving tacrolimus were administered 0.5 mg/kg intraperitoneally daily throughout the treatment period.

### Nerve Recovery Parameters

#### Electrophysiology

An electrophysiology system (ADI Powerlab 4/35 with signal filter, Colorado Springs, Colorado) optimized for small animal studies was used for compound muscle action potential (CMAP) recordings. The CMAPs were measured in the intrinsic foot muscles on the plantar surface using standard sub-dermal needle electrodes. All experiments were performed under general anesthesia (isoflurane 1.5–2.5%/O_2_) with the rats monitored for respiratory distress. Recorded maximal amplitude and latency data normalized to the contralateral, non-operated, limb was obtained. Amplitude was measured as the maximal deflection from baseline, and latency was measured as the time from stimulation to the response onset. Of note, when recording CMAPs, the stimulating mode was set as pulse mode (stimulus intensity 200 mv, frequency 4 Hz, duration 0.2 ms). Serial CMAP measurements were performed at 0, 4, 8, 12, and 16 weeks. All these measurements were performed in a blinded fashion.

#### Computer-assisted Gait Analysis

Using the Catwalk XT System (Noldus Information Technology, Leesburg, Virginia), we conducted advanced gait analysis on all animals on a bi-weekly basis. The system consists of an enclosed walkway, a high-speed color camera, and recording and analysis software to assess the locomotor performance of rodent models. While animals traverse the walkway from one side to the other side in a non-enforced manner, their footprints are captured with a high-speed video camera. The video camera sends the capture to a computer that runs the CatWalk XT software. Utilizing Illuminated Footprint technology, the paw print area, contact intensity, swing speed and swing distance are captured. From this data, numerous parameters are calculated for qualitative and quantitative analysis of individual footfalls and gait. A detailed description of the system can be found elsewhere^71^. We normalized data by dividing the operated right limb by the non-operated left limb. In order to provide consistent and reproducible functional outcome data, we introduced pre-operative training of the rats on the CatWalk system. As a result, their walk through the CatWalk walkway became unforced, continuous and consistent, leading to greater accuracy in the classification of their walking behavior. Each animal was tested for 5 runs at each time point. In addition, the calibration settings of the CatWalk were optimized to increase the detection accuracy of the machine. Pilot experiments were conducted involving various models of rat peripheral nerve injuries (sham, nerve gap, crush injury, transection and repair) to confirm reproducibility of data. After optimization, functional gait analysis was performed in a blinded fashion to compare experimental groups. Three runs with at least four step cycles were analyzed per animal. Ratios between the operated hind limn and contralateral non-operated hindlimb were calculated and the data was expressed as mean ± SD.

#### Nerve Histomorphometry

Histomorphometric measures of entire nerve cross-sections were evaluated to quantify the extent of nerve regeneration. Briefly, harvested nerves were fixed in 3% EM grade glutaraldehyde at 4 °C, post-fixed with 1% osmium tetroxide and serially dehydrated in ethanol. Specimens were then embedded in Araldite 502 (Polysciences Incorporated, Warrington, Pennsylvania), and cut into 5-μm cross-sections using an ultramicrotome. Sections at 5–8 mm distal to the anastomosis site were mounted on slides and stained with 1% toluidine blue dye for imaging. At 1000x magnification, a blinded reviewer selected 5–7 randomly selected fascicle fields per nerve fiber were evaluated for myelinated axon counts; whole nerve fiber area was also calculated. From these data, the total number of myelinated axons, nerve axon density (axons/mm^2^), average fiber diameter (μm), and average axon diameter (μm) were derived. To better elucidate the differences in early and late phases of nerve regeneration, sub-groups of experimental and control data from the sciatic nerve transection and repair study were collected at 16 weeks. For syngeneic and allogeneic groups, nerve sections were collected at 16 weeks or at the time of euthanasia, if rejection occurred prior to the 16-week time point. An investigator blinded to experimental groups measured a minimum of 300 fibers per nerve. ImageJ (National Institutes of Health, Bethesda, Maryland) was used for quantification analysis.

#### Masson’s Trichrome Staining

To quantify collagen deposition at the repair site, Masson’s trichome staining was performed by embedding the nerves in epozy resin; 2.5 μm sections were collected on coated slides and dried on a hot plate. Sections were wetted with distilled water and stained on a hot plate in the following order with warm tap water rinses in between: ferric chloride for 3 minutes, Mayer’s Hematoxylin for 25 seconds, and Ponceau-acid fuchsin for 8 minutes. Sections were rinsed with 0.5% acetic acid and then stained on a hot plate with 0.5% phosphomolybdic acid for 3 minutes and Light Green SF Yellowish for 3 min, with 0.5% acetic acid rinses in between. For each specimen, 10–12 section were taken from across the width of the nerve at the site of the neurorrhaphy. Image J (National Institutes of Health, Bethesda, Maryland) was used to outline the intraneural tissue (excluding epineurium).

### Statistical Analysis

Results are expressed as mean ± SD (standard deviation) for continuous variables. For electrophysiological parameters, unpaired two-tailed Student’s t-tests were performed to compare the mean normalized amplitudes and latencies between control and experimental groups. For gait-analysis parameters, unpaired two-tailed Student’s t-tests with Levene’s testing were performed to compare the normalized run parameters between groups. Between-group means were assessed for statistically significant differences using one-way ANOVA followed by two-tailed, two-sample, independent Student’s t-tests and the Bonferroni correction to adjust for multiple comparisons. For nerve histomorphometry, unpaired two-tailed Student’s t-tests were used to compare the axon counts, axon density, axon diameter, and nerve fiber diameter between experimental groups.

*A priori* sample size and power calculations based on previous results from similar experiments^72^,^73^ were performed to detect a minimum 3-fold increase of mean axon counts, a 50% increase in mean normalized CMAP amplitude, and a 50% reduction in mean normalized CMAP latency in the MSC-treated rats at the familywise error rate of 5% and 80% power.

A p-value of <0.05 was considered to be significant in all analyses. All analyses were performed using SPSS statistical analysis software (IBM Corporation, Armonk, New York).

## Additional Information

**How to cite this article**: Cooney, D. S. *et al.* Mesenchymal Stem Cells Enhance Nerve Regeneration in a Rat Sciatic Nerve Repair and Hindlimb Transplant Model. *Sci. Rep.* **6**, 31306; doi: 10.1038/srep31306 (2016).

## Supplementary Material

Supplementary Information

## Figures and Tables

**Table 1:** Sciatic Nerve Transection and Repair Experimental Groups.

Group (n = 4)	Experiment	Endpoint
Sciatic nerve transection and repair	No treatment	16 weeks
Sciatic nerve transection and repair	Local MSC	16 weeks
Sciatic nerve transection and repair	Systemic MSC	16 weeks

Local MSC treatment consisted of 5 × 10^4^ mesenchymal stem cells (MSCs) (reconstituted to a 5 μl volume) injected into the epineurium of the distal nerve stump, while systemic MSC treatment consisted of an IV injection of 1 × 10^6^ MSCs at the end of the surgical procedure.

**Table 2:** Syngeneic and Allogeneic Hindlimb Transplant Experimental Groups.

Donor/Recipient	Group (n = 4)	Experiment	Endpoint
Lewis/Lewis Hindlimb transplantation	SYN-Control	No treatment	Banff grade III rejection
	SYN-Control + Tacrolimus	Tacrolimus	
	SYN-Local + Tacrolimus	Local MSC + Tacrolimus	
	SYN-Systemic + Tacrolimus	Systemic MSC + Tacrolimus	
Brown Norway/Lewis Hindlimb Transplantation	ALLO-Control	No treatment	Banff grade III rejection
	ALLO-Control + Tacrolimus	Tacrolimus	
	ALLO-Local + Tacrolimus	Local MSC + Tacrolimus	
	ALLO-Systemic + Tacrolimus	Systemic MSC + Tacrolimus	

Local MSC treatment consisted of 5 × 10^4^ mesenchymal stem cells (MSCs) (reconstituted to a 5 μl volume) injected into the epineurium of the distal nerve stump, while systemic MSC treatment consisted of an IV injection of 1 × 10^6^ MSCs at the end of the surgical procedure. Tacrolimus treatment consisted of 0.5 mg/kg tacrolimus daily for the first 30 postoperative days.
