# Supplementary material for: Mesenchymal Stem Cells Enhance Nerve Regeneration in a Rat Sciatic Nerve Repair and Hindlimb Transplant Model
Source: Sci Rep. 2016 Aug 11;6:31306. doi: 10.1038/srep31306 (PMC4980673; doi:10.1038/srep31306)

Mesenchymal Stem Cells Enhance Nerve Regeneration in a Rat Sciatic Nerve Repair and Hindlimb Transplant Model

Damon S. Cooney MD PhD[1], Eric G. Wimmers MD[1], Zuhaib Ibrahim MD[1], Johanna Grahammer MD[1], Joani M. Christensen MD[1], Gabriel A. Brat, MD[1], Lehao W. Wu MD[1], Karim A. Sarhane MD[1], Joseph Lopez MD MBA[1], Christoph Wallner MD[1], Georg J. Furtmüller MD[1], Nance Yuan MD[1], John Pang MD[1], Kakali Sarkar PhD[1], W. P. Andrew Lee, MD[1], Gerald Brandacher MD[1*]

[1]Department of Plastic and Reconstructive Surgery, Johns Hopkins University School of Medicine, Baltimore, Maryland, USA,

SUPPORTING FIGURE LEGENDS

Supporting Figure 1. BM-MSC characterization. BM-MSCs consistently and homogenously expressed cell surface markers CD29 and CD90 and were negative for CD45, CD11, RT1A and RT1B as determined by flow cytometry analysis using fluorescent labeled monoclonal antibodies.

Supporting Figure 2. Differentiation Potential of BM-MSCs. (A) Chondrogenesis: arrow indicates blue-stained collagen with Alcian-Blue staining. (B) Osteogenesis: cytoplasmic calcium deposition stains black with Von Kossa staining (C) Adipogenesis: Oil-O Red staining shows red intracellular lipid-filled vesicles.

Supporting Figure 3. Representative images from sciatic nerve transection and repair rats. Treatment with (A) systemic MSC therapy, (B) local MSC therapy and (C) no treatment control. Light microscopic image (100X, stained with toluidine blue). Nerve cross sections were obtained 5-8 mm distal to anastomosis site.

Supplementary Figure 1

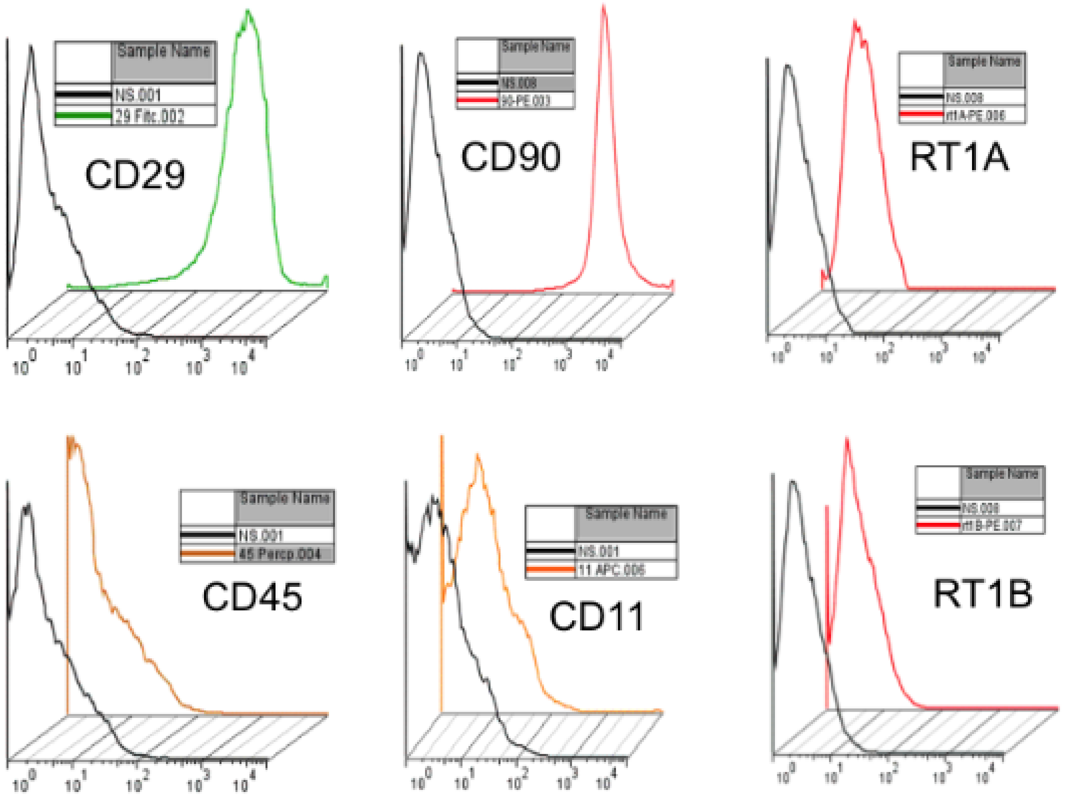

Supplementary Figure 2

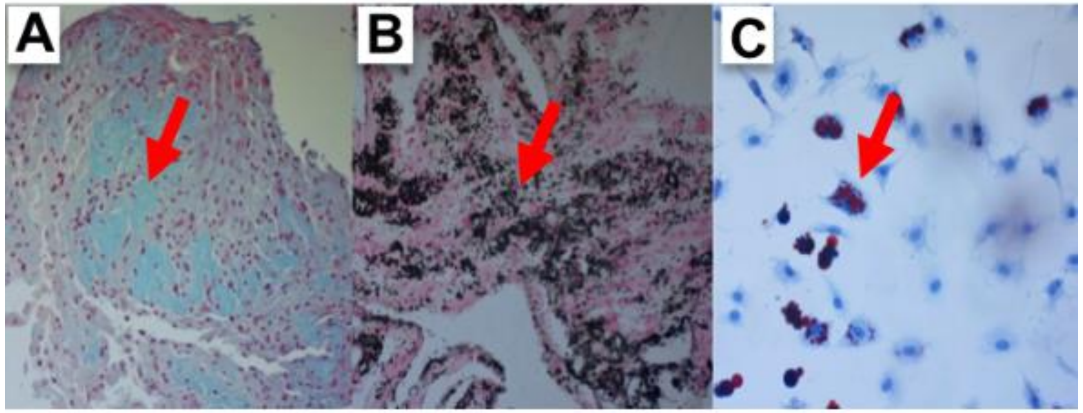

Supplementary Figure 3

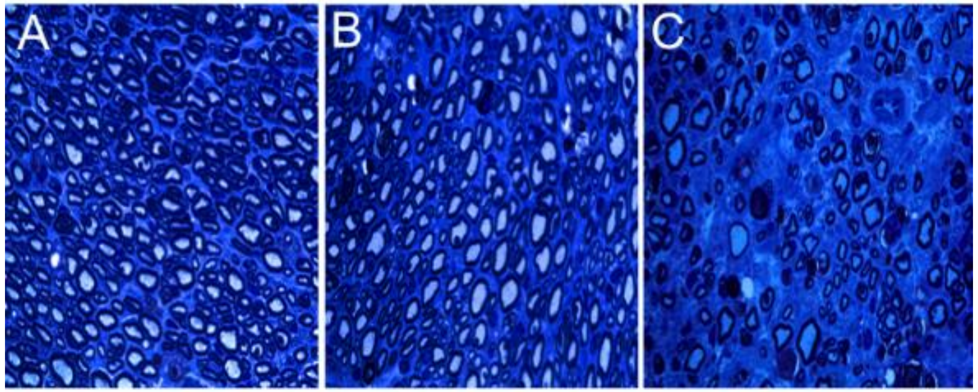

Supplement: Supplementary Information [file srep31306-s1.pdf]
